# Genomic profiling of the transcription factor Zfp148 and its impact on the p53 pathway

**DOI:** 10.1038/s41598-020-70824-2

**Published:** 2020-08-25

**Authors:** Zhiyuan V. Zou, Nadia Gul, Markus Lindberg, Abdulmalik A. Bokhari, Ella M. Eklund, Viktor Garellick, Angana A. H. Patel, Jozefina J. Dzanan, Ben O. Titmuss, Kristell Le Gal, Inger Johansson, Åsa Tivesten, Eva Forssell-Aronsson, Martin O. Bergö, Anna Staffas, Erik Larsson, Volkan I. Sayin, Per Lindahl

**Affiliations:** 1 Wallenberg Laboratory, Department of Molecular and Clinical Medicine, Institute of Medicine, University of Gothenburg, Gothenburg, Sweden; 2 Sahlgrenska Cancer Center, Department of Surgery, Institute of Clinical Sciences, University of Gothenburg, Gothenburg, Sweden; 3 Wallenberg Centre for Molecular and Translational Medicine, University of Gothenburg, Gothenburg, Sweden; 4 Department of Biochemistry, Institute of Biomedicine, University of Gothenburg, Gothenburg, Sweden; 5 Sahlgrenska Cancer Center, Department of Radiation Physics, Institute of Clinical Sciences, University of Gothenburg, Gothenburg, Sweden; 6 Department of Medical Physics and Biomedical Engineering, Sahlgrenska University Hospital, University of Gothenburg, Gothenburg, Sweden; 7 Sahlgrenska Cancer Center, Department of Molecular and Clinical Medicine, Institute of Medicine, University of Gothenburg, Gothenburg, Sweden; 8 Department of Biosciences and Nutrition, Karolinska Institute, Huddinge, Sweden; 9 Department of Microbiologi and Immunology, Institute of Biomedicine, University of Gothenburg, Gothenburg, Sweden

**Keywords:** Cell division, Computational biology and bioinformatics, Senescence

## Abstract

Recent data suggest that the transcription factor Zfp148 represses activation of the tumor suppressor p53 in mice and that therapeutic targeting of the human orthologue ZNF148 could activate the p53 pathway without causing detrimental side effects. We have previously shown that *Zfp148* deficiency promotes p53-dependent proliferation arrest of mouse embryonic fibroblasts (MEFs), but the underlying mechanism is not clear. Here, we showed that *Zfp148* deficiency downregulated cell cycle genes in MEFs in a p53-dependent manner. Proliferation arrest of *Zfp148*-deficient cells required increased expression of ARF, a potent activator of the p53 pathway. Chromatin immunoprecipitation showed that Zfp148 bound to the *ARF* promoter, suggesting that Zfp148 represses *ARF* transcription. However, Zfp148 preferentially bound to promoters of other transcription factors, indicating that deletion of *Zfp148 *may have pleiotropic effects that activate ARF and p53 indirectly. In line with this, we found no evidence of genetic interaction between *TP53* and *ZNF148* in CRISPR and siRNA screen data from hundreds of human cancer cell lines. We conclude that *Zfp148* deficiency, by increasing *ARF* transcription, downregulates cell cycle genes and cell proliferation in a p53-dependent manner. However, the lack of genetic interaction between *ZNF148* and *TP53* in human cancer cells suggests that therapeutic targeting of ZNF148 may not increase p53 activity in humans.

## Introduction

Activation of the tumor suppressor p53 may have beneficial effects on tumors with wild-type p53^1^. The first drugs targeting the p53 repressor murine double minute 2 (MDM2) have entered clinical trials, but their clinical benefit is still under evaluation^2^. Because knockout of *Mdm2* in mice causes widespread and lethal activation of p53 in normal tissues^3^, efficient pharmacological inhibition of MDM2 may have adverse effects. Thus, identifying alternative ways to activate the p53 pathway with less adverse effects is warranted.

Zinc finger protein 148 (Zfp148, Zbp-89, BFCOL, BERF1, htβ) is a transcription factor that binds to GC-rich DNA sequences, thus activating or repressing transcription of target genes^4–11^. Earlier studies have shown that Zfp148 is a potent repressor of p53 activity in mice. Firstly, deletion of *Zfp148* caused p53-dependent proliferation arrest of cultured mouse embryonic fibroblasts (MEFs) and prenatal lung tissue that was rescued by reducing oxidative stress^12^. Moreover, deletion of one copy of *Zfp148* in the *Apc*^*Min/*+^ model of intestinal adenomas reduced tumor numbers and increased survival by increasing p53 activity^13^. In line with this, conditional deletion of one or both alleles of *Zfp148* in the gut epithelium of *Apc*^*FL/*+^ mice reduced tumor formation through a mechanism involving β-catenin^14^. Finally, deletion of one copy of *Zfp148 *reduced proliferation of macrophages and atherosclerosis in *Apoe*^−*/*−^ mice by increasing p53 activity^15^. These results indicate that deletion of one copy of *Zfp148* is sufficient to induce therapeutically meaningful p53 activation. This is important because mice lacking one copy of *Zfp148* have a normal life span and appear to be healthy. Therapeutic targeting of Zfp148 could therefore protect against cancer or atherosclerosis by increasing p53 activity without causing detrimental side effects.

The mechanism leading to increased p53 activity is not well understood. Zfp148 interacts physically with p53^16^ raising the possibility that Zfp148 regulates p53 by protein–protein interaction. However, indirect mechanisms are equally possible. Here, we investigate mechanisms behind cell cycle arrest of *Zfp148*-deficient (*Zfp148*^*gt/gt*^) MEFs by using global gene expression analysis, targeted inactivation of *Cdkn2a* genes, genome-wide chromatin immunoprecipitation, and public CRISPR and siRNA screen data.

## Results

### *Zfp148* deficiency downregulates expression of E2F-responsive cell cycle genes

**Figure 1:**
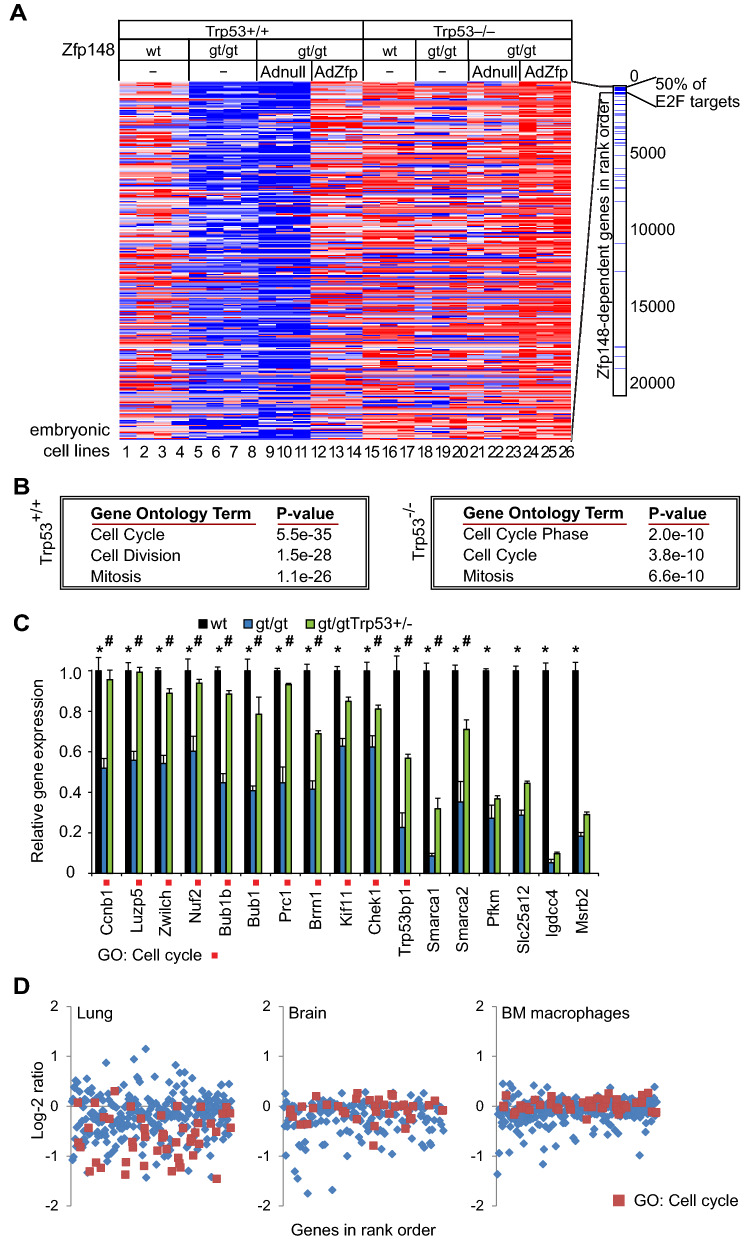
E2F-responsive cell cycle genes are downregulated in *Zfp148*-deficient cells. (**A**) Heat-map showing expression profiles of the top 300 genes that were downregulated in the absence of Zfp148 (blue colour indicates downregulation, red indicates upregulation). Bar showing the rank order of 59 validated E2F target genes according to the heat-map. (**B**) Table showing enriched GO terms on *Trp53*^+*/*+^ (left) or *Trp53*^−/−^ (right) genetic background in rank order. (**C**) Real-time RT-PCR analysis of 17 genes selected from the top 300-set in wild-typeand *Zfp148*^*gt/gt*^ MEFs on *Trp53*^+*/*+^ (n = 3) or *Trp53*^+/−^ (n = 4) genetic background. Red box indicates genes linked to the GO-term “Cell Cycle”. * and # indicate significant differential expression in wild-type versus *Zfp148*^*gt/gt*^ and *Zfp148*^*gt/gt*^ versus *Zfp148*^*gt/gt*^*Trp53*^+*/−*^ MEFs, respectively. (**D**) Graphs showing log2 expression ratios of the top-300 downregulated genes in lungs (left), brains (middle), and bone marrow-derived macrophages (right) from newborn *Zfp148*^*gt/gt*^ mice compared to controls (n = 3). Red colour indicates genes linked to the GO term “Cell Cycle”.

To define mechanisms that cause proliferation arrest of *Zfp148*^*gt/gt*^ MEFs, we used transcript profiling to identify genes that were differentially expressed between *Zfp148*^*gt/gt*^ MEFs and wild-type controls. We also compared *Zfp148*^*gt/gt*^ MEFs infected with adenovirus carrying *Zfp148* cDNA or empty vector to verify that differentially expressed genes depended on Zfp148. The analysis revealed a set of more than 300 genes that were downregulated in *Zfp148*^*gt/gt*^ MEFs compared to wild-type controls, and rescued in *Zfp148*^*gt/gt*^ MEFs after adenoviral expression of *Zfp148* cDNA compared to empty vector (Fig. 1A and Supplementary Data S1). Overrepresentation analysis showed strong enrichment of cell cycle-related genes (Fig. 1B). Since this category of genes is regulated by E2F transcription factors, we identified known E2F targets from earlier publications^17,19^ and investigated their rank in the gene list. The majority of known E2F targets clustered at the top of the list confirming that *Zfp148* deficiency downregulated E2F-dependent cell cycle genes (Fig. 1A).

To investigate the impact of p53 on gene expression, we repeated the analysis with RNA extracted from MEFs generated on a *Trp53*-null genetic background. Downregulation of the cell cycle-enriched gene set was markedly attenuated on this background (Fig. 1A), confirming that p53 plays a dominant role. However, the rank of differentially expressed genes was similar to the rank obtained on *Trp53* wild-type background (Spearman correlation, r = 0.56, *P* < 0.0001) with strong enrichment of cell cycle-related gene ontology (GO) terms among the downregulated genes (Fig. 1B). We therefore conclude that *Zfp148* deficiency downregulates cell cycle genes by both p53-dependent and p53-independent mechanisms.

The previous data panels show qualitative changes. To obtain quantitative data, we determined mRNA levels with real-time RT PCR for 17 genes arbitrarily selected from the downregulated gene set. Expression of genes related to the GO term cell cycle was reduced by half in *Zfp148*^*gt/gt*^ MEFs compared to controls (Fig. 1C). Deletion of one copy of *Trp53* is sufficient to rescue cell proliferation of *Zfp148*-deficient MEFs^12^. In line with this, expression of 11 genes was restored to near normal levels in *Zfp148*^*gt/gt*^*Trp53*^+*/−*^ compared to *Zfp148*^*gt/gt*^ MEFs, and 9 of those genes were linked to the GO-term cell cycle (Fig. 1C). Only 1 of the 6 remaining genes that were not restored on *Trp53*^+*/−*^ background was linked to the cell cycle. These results suggest that p53-mediated repression of cell cycle genes causes the proliferative arrest of *Zfp148*^*gt/gt*^ MEFs.

To assess whether downregulation of the gene set observed in response to *Zfp148* deficiency in MEFs also occurred in vivo, we isolated RNA from neonatal lungs and brains and from bone marrow-derived macrophages of *Zfp148*^*gt/gt*^ and wild-type mice. We determined gene expression by microarrays and showed that the gene set was markedly downregulated compared to wild-type controls in *Zfp148*^*gt/gt*^ lungs but not in brains or macrophages (Fig. 1D). Previous studies showed that *Zfp148* deficiency induces cell cycle arrest in prenatal lungs in a p53-dependent manner^12^. In contrast, brains and bone marrow derived macrophages from *Zfp148* deficient mice displayed no overt phenotypes^12,20,21^. Thus, the observed downregulation of cell cycle genes in lungs but not brain or bone marrow derived macrophages from *Zfp148* deficient mice compared to controls makes sense (Fig. 1D).

### The *Cdkn2a* transcript *ARF* is required for cell proliferation arrest of *Zfp148*^*gt/gt*^ MEFs

**Figure 2:**
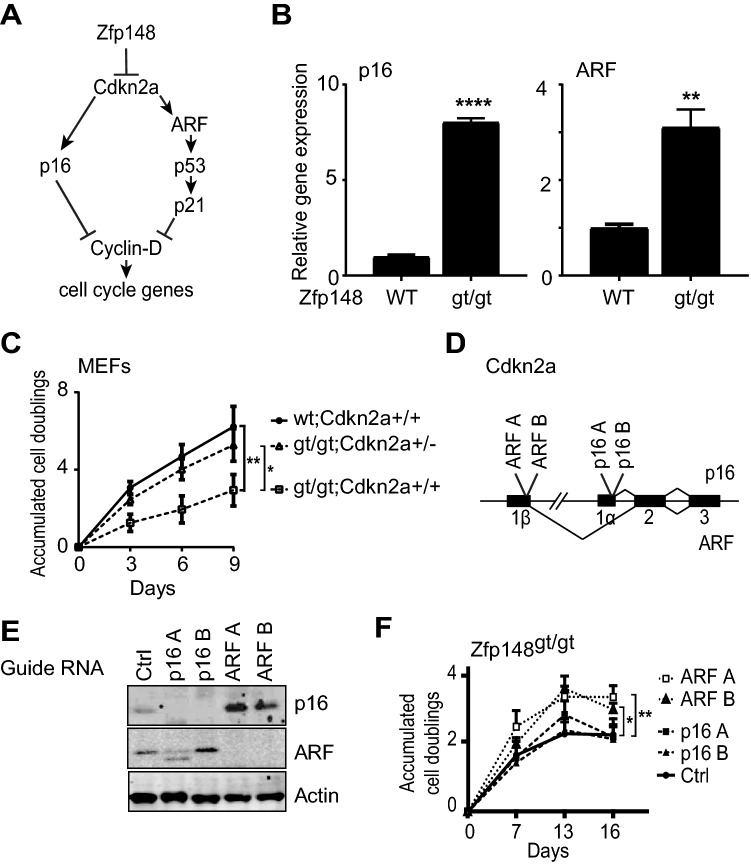
The *Cdkn2a* gene product ARF reduces proliferation of *Zfp148*^*gt/gt*^ MEFs. (**A**) Schematic of *Cdkn2a* regulation of cell cycle genes. (**B**) Graphs showing *p16* and *ARF* mRNA levels in wild-type and *Zfp148*^*gt/gt*^ MEFs (n = 6). (**C**) Growth curves (accumulated cell doublings) of wild-type MEFs and *Zfp148*^*gt/gt*^ MEFs on *Cdkn2a*^+*/*+^ and *Cdkn2a*^+*/−*^ background (n = 5). (**D**) Schematic of the mouse *Cdkn2a* locus showing genome locations of guide-RNA target sites for ARF A, ARF B, p16 A, and p16 B. (**E**) Western blots showing expression of ARF and p16 in *Zfp148*^*gt/gt*^ MEFs infected with guide-RNAs targeting *ARF*, *p16*, or scrambled control. Actin was used as loading control. Full-length blots are presented in Supplementary Fig. S1. (**F**) Graphs showing growth (accumulated cell doublings) of *Zfp148*^*gt/gt*^ MEFs infected with the indicated guide-RNAs (n = 5, data are mean ± SEM). *P < 0.05, **P < 0.01, ***P < 0.001.

The downregulation of cell cycle genes in *Zfp148*^*gt/gt*^ MEFs by p53-dependent and -independent mechanisms prompted us to investigate mRNA levels of the cyclin-dependent kinase inhibitor *Cdkn2a*. *Cdkn2a* produces two gene products, ARF and p16, which downregulate cell cycle genes by activating the p53 pathway and inactivating the CDK4/6 complex, respectively (Fig. 2A). *ARF* and *p16* mRNA were markedly increased in *Zfp148*^*gt/gt*^ MEFs compared to controls (Fig. 2B), supporting a role for *Cdkn2a* in the downregulation of cell cycle genes.

We have previously demonstrated that *Zfp148* deficiency induces cell proliferation arrest in MEFs after a few passages, and that p53 is required for the arrest^12^. To further investigate the role of *Cdkn2a* in the proliferation arrest, *Zfp148*^*gt/*+^ mice were bred on a *Cdkn2a*^+*/−*^ background to generate *Zfp148*^*gt/gt*^*Cdkn2a*^+*/−*^ MEFs. Deletion of one copy of *Cdkn2a* restored normal proliferation of *Zfp148*^*gt/gt*^ MEFs (Fig. 2C), demonstrating that *Cdkn2a* is required for proliferation arrest of these cells. To determine the relative importance of ARF and p16 for the proliferation arrest, we used lentiviral guide-RNAs and CRISPR to generate *Zfp148*^*gt/gt*^ MEFs lacking either ARF or p16 (Fig. 2D, E and Supplementary Fig. S1). Selective *ARF* knockout rescued growth of *Zfp148*^*gt/gt*^ cells (Fig. 2F) despite increased expression of p16, which is an expected consequence of ARF inactivation^22^. Knockout of *p16* had no effect on cell growth (Fig. 2F). Collectively, these data show that ARF but not p16 is required for p53 activation and proliferation arrest of *Zfp148*^*gt/gt*^ MEFs.

### Zfp148 binds to clustered cytosine-rich DNA elements in basal promoters of other transcription factors

To understand the primary basis of ARF and p53 activation in *Zfp148*^*gt/gt*^ MEFs, we used chromatin immunoprecipitation-on-chip (ChIP-chip) assays to identify target sites of FLAG-tagged Zfp148 (AdZfp148FLAG) in *Zfp148*^*gt/gt*^*Trp53*^−/−^ MEFs. The experiment was performed on a *Trp53*-null background to overcome senescence and obtain sufficient amounts of starting material. The CMV-driven adenovirus vector used produces Zfp148 protein amounts that are similar to those found in wild-type MEFs^12^.

**Figure 3:**
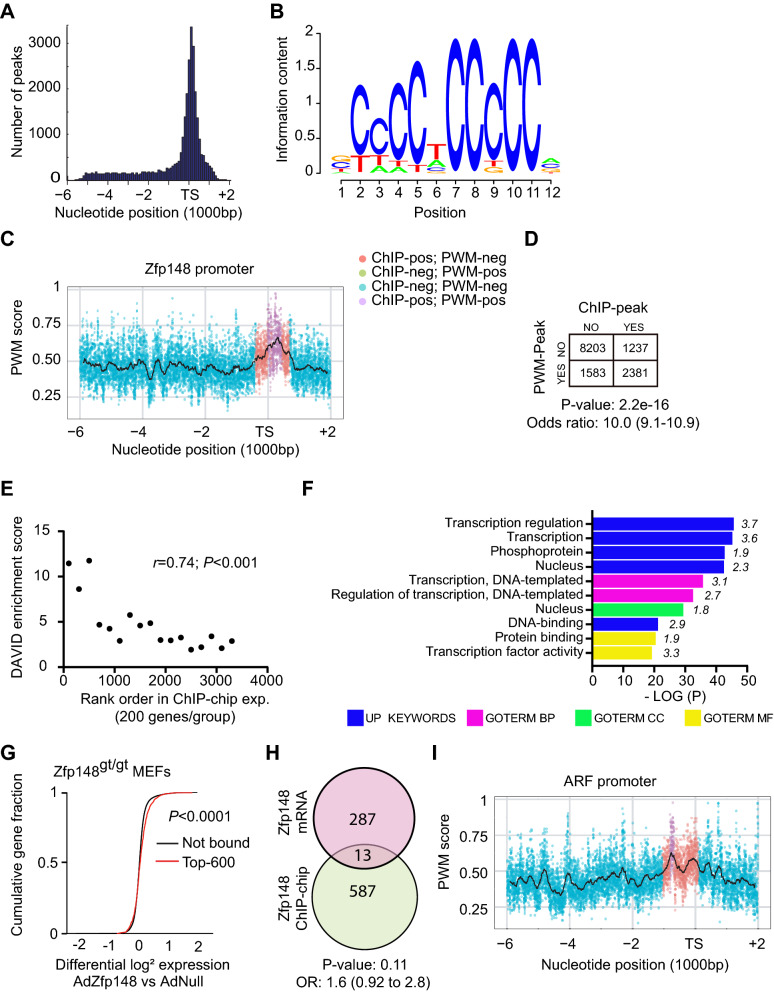
Zfp148 binds to clustered cytosine-rich elements in promoters of other transcription factors. (**A**) Frequency plot showing the location of binding sites of AdZfp148FLAG in relation to transcription starts (TS) for one representative ChIP-chip experiment. (**B**) Position Weight Matrix (PWM)-logo for Zfp148. (**C**) A representative gene (*Zfp148*) with both ChIP-peaks and PWM-peaks. Each point represents the PWM score at the specific position, taking only the best score of the two strands. The black line indicates the mean score over the current position in a 200 bp surrounding window. The intersect of ChIP- and PWM-peaks is indicated by a color code (explained in the figure). (**D**) Contingency table showing the distribution of promoters with or without ChIP- and/or CWM-peaks. 95% confidence interval in brackets. (**E**) Graph showing maximum DAVID enrichment score on y-axis and rank order on x-axis for bins of 200 genes based on ChIP-binding score. r indicates Spearman correlation. (**F**) Graph showing enriched functional terms for the top-600 AdZfp148FLAG binding genes. Fold-enrichment is shown in italics. − LOG10 *P*-value, negative logarithm with base 10 of the adjusted p-value; UP, UniPRot; GOTERM, gene ontology term; CC, cell compartment; MF, molecular function; BP, biological process. (**G**) Cumulative distribution function (CDF) of log2-transformed gene expression ratios in *Zfp148*^*gt/gt*^ MEFs transduced with AdZfp148 versus AdNull. Red line, genes that bind AdZfp148FLAG with high affinity (n = 600 based on rank order); black line, genes that did not bind AdZfp148FLAG in the ChIP-chip experiments (n = 10,163) (expression analysis, n = 3; ChIP-chip analysis, n = 4). (**H**) Venn diagram showing the intersect of the top-300 genes that were downregulated in Fig. 1 (red) and the top-600 AdZfp148FLAG binding genes (green). 95% confidence interval in brackets. (**I**) Graph showing ChIP scores and PWM scores for the ARF promoter. Data representation as in (**C**).

Zfp148 target sites were recorded in four independent experiments with a strong preference for binding close to transcription starts (Fig. 3A and Supplementary Data S2). To determine the DNA-binding preferences of Zfp148, we constructed a position weight matrix (PMW) model from 16 known Zfp148 binding sites (Table 1). As expected, the model predicted a cytosine-rich binding motif (Fig. 3B). We next applied the model to DNA regions surrounding known transcription starts. While there was no correlation between peak positions of ChIP-chip data and PWM-predicted single motifs, Zfp148 showed a strong preference for binding to regions with clustered PWM-predicted motifs (Fig. 3C,D). These data indicate that Zfp148 binds to clustered motifs in cytosine-rich basal promoters.

**Table 1:** Zfp148 binding sites used to construct a position weight matrix (PMW) model.

Symbol	Description	Organism	Genomic site	References
TCRB	T cell receptor B	Human	CCACCACCCCCA	Wang^5^
Ptcra	Pre-T cell receptor A	Mouse	ACCCCACCCCCA	Wang^5^
Ptcra	Pre-T cell receptor A	Mouse	GCCCCTCCCCCG	Reizis and Leder^42^
LCK	Lymphocyte-specific tyrosine kinase	Human	TCACCACCCCCA	Yamada^43^
MMP3	Matrix metalloproteinase 3	Human	TTTTTTCCCCCC	Ye^44^
Col1a1	Collagen type I alpha 1	Mouse	CCTCCTCCCCCC	Hasegawa^6^
Col1a1	Collagen type I alpha 1	Mouse	GCCCCCCCTCCC	Hasegawa^6^
Col1a2	Collagen type I alpha 2	Mouse	GTCCCTCCCCCC	Hasegawa^6^
PDGFRA	Platelet-derived growth factor receptor, alpha	Human	TCCCCTCCCCCG	De Bustos^45^
ODC1	Ornithine decarboxylase	Human	GCCCCTCCCCCG	Law^46^
CDKN1A	Cyclin-dependent kinase inhibitor 1A	Human	CCTCCTCCCCCA	Bai^8^
STAT1	Signal transducer and activator of transcription 1	Human	GTCCCACCCCCG	Bai^47^
GAS	Gastrin	Human	CCCACCCCGCCC	Merchant^4^
CXCL5	Chemokine, cxc motif, ligand 5	Human	CCCCCTCCCCCA	Keates^48^
ENO3	Beta enolase	Human	CCCCCTCCCCCA	Feo^49^
Adx1	Adrenodoxin	Bovine	GCCCCGCCCCCT	Cheng^50^

Overrepresentation analysis of 3,488 genes that bound to Zfp148 at 1% false discovery rate in all experiments showed strong enrichment of functional terms. Binning genes according to their rank in the ChIP-chip experiment revealed a striking correlation between binding scores and the enrichment of functional terms; the enrichment declined abruptly after 600 genes (Spearman correlation r = 0.74, *P* < 0.001, Fig. 3E). Since functional connectivity is an expected property of co-regulated genes, the number of Zfp148 target genes is likely much lower than the number of binding genes. The top-ranked genes (top 600) were enriched for functional terms related to transcriptional regulation, suggesting that Zfp148 mainly operates upstream of other transcription factors (Fig. 3F). There was no overrepresentation of cell cycle-related terms.

By integrating ChIP-chip and gene expression data, we confirmed a small but significant upregulation of Zfp148 target genes in *Zfp148*^*gt/gt*^ MEFs after infection with AdZfp148 (Fig. 3G). However, there was no significant overlap between Zfp148-binding genes and the set of cell cycle-enriched genes that was downregulated in *Zfp148*^*gt/gt*^ MEFs (Fig. 3H). Notably, Zfp148 bound to the *ARF* promoter in all experiments with binding regions centered at 800 base pairs upstream of the transcription start (Fig. 3I) suggesting that Zfp148 may repress *ARF* transcription. However, the preferential binding of Zfp148 to promoters of other transcription factors indicates that deletion of *Zfp148* could have pleiotropic effects that activate ARF and p53 indirectly.

### No genetic interaction between *ZNF148* and *TP53* in human cancer cell lines

Since the integrated transcriptomic and promoter occupancy analysis suggested pleiotropic and indirect mechanisms of p53 and ARF activation in *Zfp148*^*gt/gt*^ MEFs, we decided to explore the genetic interaction between *ZNF148* (the human orthologue of Zfp148) and *TP53* in CRISPR and siRNA data from human cancer cell lines.

**Figure 4:**
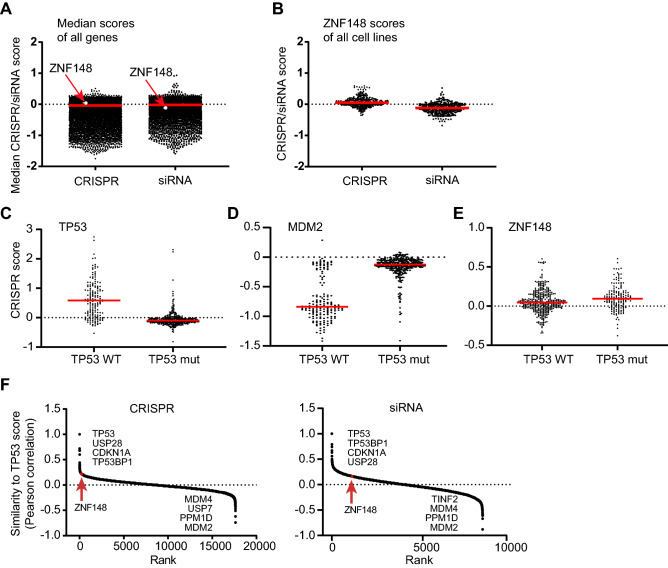
No genetic interaction between *ZNF148* and *TP53* in human cancer cell lines. (**A**) Graph showing the median CRISPR and siRNA scores across the human cancer cell lines for all genes. *ZNF148* scores are indicated by arrows. n = 17,631 (CRISPR) and 16,725 (siRNA) genes, respectively. (**B**) Graph showing the CRISPR and siRNA scores of *ZNF148* for all cancer cell lines. n = 432 (CRISPR) and 328 (siRNA) cell lines, respectively. (**C**–**E**) Graphs showing the CRISPR scores of *TP53* (**C**) or *MDM2* (**D**) or *ZNF148* (**E**) for cell lines with wild-type (left, n = 354) or mutated (right, n = 164) *TP53*. (**F**) Graphs showing Pearson correlation with CRISPR (left) or siRNA (right) scores of *TP53* (y-axis) for all genes sorted by rank (x-axis). The position of *ZNF148* is indicated by arrows. n = 17,631 (CRISPR) and 8,296 (siRNA) genes, respectively.

We downloaded CRISPR and siRNA scores for all genes in 487 human cancer cell lines from the Cancer Dependency Map database (https://depmap.org). A negative score indicates that knockout (CRISPR) or knockdown (siRNA) of that gene has a negative impact on cell growth or survival. Genes with CRISPR scores below − 0.6 are considered to be essential^23^. The median CRISPR and siRNA scores for *ZNF148* were 0.05 (± 0.01 95% CI) and − 0.12 (± 0.02 95% CI), suggesting that cancer cells are marginally affected by *ZNF148* inactivation (Fig. 4A). *ZNF148* was not essential for the growth or survival of any of the tested cell lines (Fig. 4B).

We next divided the cell lines into two groups depending on their *TP53* genotype. As expected, inactivation of *TP53* increased the growth of cells with wild-type *TP53* but had no impact on cells with *TP5*3 mutations (Fig. 4C). The p53 repressor *MDM2* showed a reverse pattern with low scores in *TP53* wild-type cells and scores near zero in cells with *TP53* mutations (Fig. 4D). In contrast, there was no marked difference in *ZNF148* scores between *TP53* wild-type and mutant cell lines (Fig. 4E).

We finally ranked the genes according to their similarity with *TP53* as judged by the Pearson correlation of CRISPR or siRNA scores across the set of human cancer cell lines. Canonical activators of the p53 pathway such as *USP28*, *CDKN1A*, and *TP53BP1* showed strong correlation with *TP53* and were positioned at the top of the list, while repressors including *MDM2, PPM1D*, and *MDM4* were positioned at the end (Fig. 4F). In contrast, *ZNF148* did not show strong correlation or anti-correlation with *TP53* (Fig. 4F), suggesting that there is no genetic interaction between *ZNF148* and *TP53* in human cancer cell lines under standard culture conditions.

**Figure 5:**
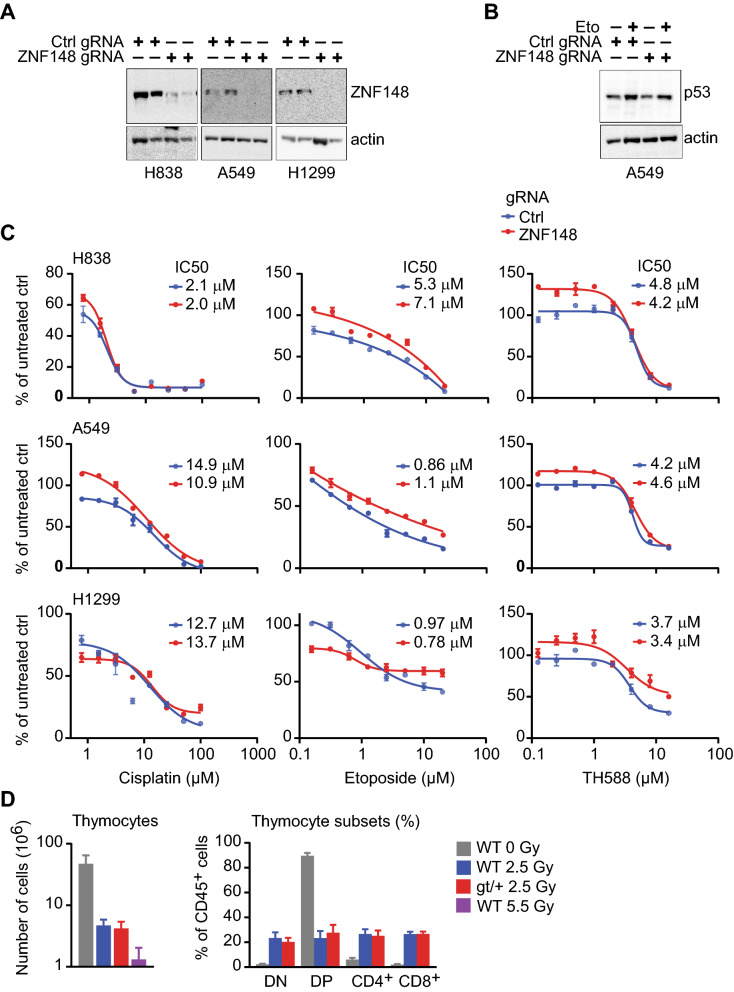
*ZNF148* deficiency does not sensitize cells to DNA damage. (**A**) Protein extracts of H838, A549, and H1299 cells expressing gRNA targeting *ZNF148* or non-targeting control, blotted with an antibody against ZNF148. Actin was used as loading control. (**B**) Protein extracts of etoposide (3uM) or vehicle treated A549 cells expressing gRNA against *ZNF148* or non-targeting control, blotted with an antibody against p53. Actin was used as loading control. Full-length blots of (**A**) and (**B**) are presented in Supplementary Fig. S3. (**C**) Drug response curves of H838, A549, and H1299 cells expressing gRNA targeting *ZNF148* or non-targeting control, for cisplatin, etoposide, or TH588 (n = 3 replicates per concentration). IC50, the half maximal inhibitory concentration. (**D**) Graphs showing total number of thymocytes (left), or thymocyte subsets as percent of CD45^+^ cells (right), in wild-type (WT) or *Zfp148*^*gt/*+^ (gt/+) mice exposed to whole body irradiation at 0, 2.5, or 5 Gy (n = 2 WT at 0 Gy, 12 WT and 7 gt/+ at 2.5 Gy, 4 WT at 5 Gy). DP, double CD4- and CD8-positive cells; DN, double CD4- and CD8-negative cells; error bars, S.E.M.

To exclude the possibility that ZNF148 exerts mild regulation of p53 that is compensated under standard conditions, we investigated the role of ZNF148 under DNA damaging conditions. We used CRISPR-Cas9 to delete *ZNF148* in H838, A549, and H1299 (*TP53* null) human lung cancer cells (Fig. 5A and Supplementary Fig. S3), and confirmed that p53-induction by etoposide was intact (Fig. 5B and Supplementary Fig. S3). To uncover quantitative differences in the DNA damage response, we established dose–response curves for cisplatin and etoposide that activates p53 through ATM/ATR, and for TH588 that activates p53 though USP28^24^. There was no difference in IC50 values between *ZNF148* knockout cells and controls in any of the tested cell lines (Fig. 5C).

To assess the role of Zfp148 under DNA damaging conditions in vivo, we exposed *Zfp148* heterozygous mice and controls to ionizing radiation and quantified the depletion of immature thymocytes, a process that is entirely p53-dependent^25^. As expected, ionizing radiation reduced the number of thymocytes in a dose dependent manner (Fig. 5D). However, there was no difference in the total number of thymocytes, or the distribution of different thymocyte subsets, between *Zfp148*^*gt/*+^ and control mice 72 h after exposure to ionizing radiation (Fig. 5D). We conclude that Zfp148 does not regulate the p53-pathway under DNA damaging conditions.

## Discussion

We have previously shown that *Zfp148* deficiency arrests proliferation in MEFs by activating p53^12^, and here we investigated the underlying mechanism by analyzing the transcriptional output and promoter occupancy of Zfp148 in MEFs. Our results suggest that *Zfp148* deficiency, by derepressing *ARF* transcription, downregulates cell cycle genes and cell proliferation in a p53-dependent manner. The model is supported by three lines of evidence. Firstly, *Zfp148* deficiency downregulated cell cycle genes in a p53-dependent and -independent manner, with p53 playing a dominant role. Secondly, proliferation arrest of these cells required increased expression of the *Cdkn2a* transcript *ARF*, which is a major regulator of the p53 pathway. Finally, Zfp148 interacted physically with the *ARF* promoter, indicating that Zfp148 may regulate *ARF* transcription.

Zfp148 did not bind to promoters of cell cycle genes more often than predicted by chance, suggesting that regulation of cell cycle genes is indirect. p53 can downregulate cell cycle genes by inducing the cyclin-dependent kinase inhibitor p21, which is also regulated by ZNF148^8,9^. Although regulation of the *p21* promoter by ZNF148 could theoretically explain the p53-independent repression of cell cycle genes, the promoter was negatively regulated by ZNF148 (in response to butyrate) in most published experiments^8,9^, which would not explain our current finding. Given that the second transcript of the *Cdkn2a* locus, *p16*, was markedly increased in *Zfp148*^*gt/gt*^ MEFs compared to controls, a more likely explanation is that p53-independent downregulation of cell cycle genes is mediated by p16.

The cell cycle arrest in *Zfp148*^*gt/gt*^ MEFs was rescued by heterozygous deletion of *Cdkn2a* or selective knockout of the *ARF* transcript, indicating a plausible mechanism for p53 activation by ARF. In contrast to homozygous deletion of *Cdkn2a*, which causes hyper-proliferation of MEFs and spontaneous escape from senescence, heterozygous deletion does not increase cell proliferation on its own^26^. Hence, increased expression of ARF is likely part of the mechanism underlying proliferation arrest in *Zfp148*^*gt/gt*^ MEFs. We have previously shown that *Zfp148*^*gt/gt*^ MEFs are rescued by exogenous antioxidants or culture at reduced oxygen concentration showing that oxidative stress contributes to the proliferation arrest in these cells^12^. Because ARF suppresses the transcriptional activity of NRF2 (NFE2L2)^27^—the master regulator of endogenous antioxidants—increased *ARF* transcription may explain the oxidative stress weakness of *Zfp148*^*gt/gt*^ MEFs.

Zfp148 interacted physically with the *ARF* promoter, raising the possibility that *ARF* is a direct target of Zfp148. Thus, deletion of *Zfp148* may activate p53 by derepressing *ARF* transcription, similar to knockouts of polycomb members *Bmi1, M33, Mel18,* and *Phc2*^28–31^. In line with this, inhibition of ZNF148 potentiates butyrate-induced senescence of human HT116 colon cancer cells by derepressing the *p16* promoter^32^. However, increased expression of ARF could have many underpinnings. The strong enrichment of transcription factors among Zfp148-binding genes suggests that Zfp148 operates at a high level of the transcription factor hierarchy^33^. Small orchestrated alterations of many transcripts may therefore contribute to the activation of ARF and p53 in *Zfp148*^*gt/gt*^ MEFs, possibly by affecting cellular homeostasis. Collectively, our data suggest that *Zfp148* deficiency activates p53 indirectly, by derepressing *ARF* and by altering the activity of other transcription factors.

A previous publication showed that ZNF148 interacts physically with p53^16^, thus identifying another possible mechanism behind the activation of p53 in *Zfp148*^*gt/gt*^ MEFs. However, our current data on *ZNF148* and *TP53* dependencies in human cancer cells strongly argue against a physical interaction between ZNF148 and p53 playing a significant role. There was no evidence of genetic interaction between *ZNF148* and *TP53* in CRISPR and siRNA screens of hundreds of human cancer cell lines, performed under conditions that revealed strong dependencies between *TP53* and known repressors of p53 activity including *MDM2*, *MDM4*, and *PPM1D*. Moreover, the was no evidence of genetic interaction between *ZNF148* and *TP53* under DNA damaging conditions in human lung cancer cells or mice. However, it remains possible that regulation of *ARF *is important. For reasons not well understood, ARF appears to play a more prominent role as a tumor suppressor in mice than in humans^34^. Moreover, accumulating evidence indicate that ARF has important roles that are independent of p53^34^. In line with this, we observed that the correlation between *CDKN2A* and *TP53* was weak in the CRISPR screen and absent in the siRNA screen (data not shown).

In the light of these results, we need to reevaluate the role of Zfp148 and its impact on the p53 pathway. Without evidence of genetic interaction between *ZNF148* and *TP53* in human cancer cells, the incentive for therapeutic targeting of ZNF148 becomes weak. The recent finding that inhibition of ZNF148 potentiates butyrate-induced senescence of human HT116 colon cancer cells^32^ suggests that core functions of Zfp148 are evolutionarily conserved. Further investigation of the interaction between Zfp148 and *Cdkn2a* promoters may provide valuable mechanistic insights. However, transcriptomes are shaped by combinatorial interactions of transcription factors forming complex network motifs, including feedforward and feedbackward loops, that cooperatively govern gene expression^35^. Our current data suggest that Zfp148 operates at a high level of the transcription factor hierarchy. Thus, future studies of Zfp148 warrant a more integrated and comprehensive approach.

## Material and methods

### Mice

*Cdkn2a*^*tm1rdb*^ (*Cdkn2a*^−*/*^) and *Trp53*^*tm*1*Tyj*^ (*Trp53*^−*/*^) mice were obtained from The Jackson Laboratory and *Zfp148*^*gt/*+^ mice were produced by us^12^. The mice were kept on a 129/Bl6 mixed genetic background and all experiments were performed with littermate controls. Genotyping was performed by PCR amplification of genomic DNA from mouse biopsies obtained during earmarking. PCR primers used for genotyping are listed in Supplementary Table S1. Mice were fed on a regular diet and had unlimited supply of food and water. All animal procedures used in this study were approved by The Animal Research Ethics Committee in Gothenburg and performed in accordance with relevant guidelines and regulations.

### Cell culture

Primary MEFs were isolated from E13.5–15.5 embryos as described^12^. Cells were cultured in DMEM low glucose medium with 10% fetal bovine serum, 100 µg/ml penicillin and streptomycin, 1% non-essential amino acids, 1–4 mM glutamine, and 0.5 mM β-mercaptoethanol. Growth curves were established using a modified 3T3 protocol (50,000 cells/well in 6-well plates).

### Adenoviral transduction

Cells were incubated with 15 multiplicities of infection of empty control adenoviruses (Ad*Null)* or adenoviruses encoding Zfp148 (AdZfp148) or Zfp148FLAG (AdZfp148FLAG) (Vector Biolabs) for 36–48 h before analyses, as described in^12^.

### Gene expression analysis

Total RNA from MEFs, lungs, brains and macrophages was isolated using the Gene Elute kit (Sigma) and hybridized to Affymetrix Mouse Gene 1.0 ST chips. Data were normalized with the Robust Multi-Array Analysis (RMA) method^36^. The heat-map was constructed using the Hierarchical Clustering Explorer 3.0 software. Gene ontology statistics was calculated using the DAVID software^37,38^.

### Real-time quantitative PCR

TaqMan and SYBR Green assays were performed as described^39^ using TaqMan/SYBR Green universal PCR mastermix (A25741, Applied-biosystem) and the pre-designed TaqMan assays (Applied Biosystems) or RT-PCR primers listed in Supplementary Table S1.

### CRISPR-Cas9 knockouts

Pre-designed guide-RNA (gRNA) sequences targeting *p16* or *ARF *or *ZNF148* or control (does not target a sequence in the genome) were cloned into the Lenticrisprv2 vector, and co-transfected with pCMV-dR8.2 (Addgene # 8455) and pCMV-VSV-G (Addgene # 8454) vectors into HEK293T cells to produce complete Cas9 and gRNA coding lentivirus. Virus infected *Zfp148*^*gt/gt*^ MEFs or human lung cancer cells (H838, A549, or H1299) were selected with puromycin for 3 days to obtain batch clones that were used for experiments. The gRNA sequences used are listed in Supplementary Table S1. Lenticrisprv2 was a kind gift from Feng Zhang (Addgene # 52961)^40^.

### Western blot analyses

Protein levels was determined as previously described^41^ with antibodies against p16ink4a (sc-1207, santa cruz), p19^arf^ (sc-3278, santa cruz), ZNF148 (HPA001656, SigmaAldrich Atlas), p53 (DO-1 (sc126), Santa Cruz), and actin (A2066, Sigma Aldrich Atlas). Secondary antibodies were anti-mouse IRDye 680RD (926-68072, LI-COR Biosciences), anti-rabbit 680RD (926-68071, LI-COR Biosciences), anti-rabbit IgG Conformation Specific (L27A9) HRP Conjugate (5,127, Cell Signaling), and anti-mouse IgG-hl-HRP (ab6728, abcam). Protein bands were detected with the immubilon western chemiluminescent HRP substrate (Millipore) using a Chemi Doc Touch Imaging system (Bio Rad) or the Li-Cor Odyssey Imager. Exposure times were adjusted to the signal intensity of each blot. Images were adjusted for brightness and contrast with the Photoshop tool levels. All changes were applied equally across the entire image.

### ChIP-on-chip analysis

MEFs (1 × 10^8^) transduced with AdZfp148FLAG were fixed in 1% formaldehyde. 200–500-bp DNA fragments were precipitated using the anti-FLAG M2 antibody (Sigma) conjugated to sheep anti-mouse IgG Dynabeads (Invitrogen), purified (QIAquick, Qiagen), amplified (WGA2 kit, Sigma), and hybridized to MM8_Deluxe_Promoter_HX1 chips at NimblGen (www.nimblegen.com). The analysis was done on *Zfp148*^*gt/gt*^ background to avoid competition with endogenous Zfp148 and *Trp53*^−*/*−^ background to overcome senescence and obtain sufficient amounts of starting material. Gene ontology statistics was calculated using the DAVID software with “functional annotation clustering” setting for DAVID enrichment scores and “functional annotation chart” setting for ranking of functional terms^37,38^.

### Motif prediction

A position weight matrix (PWM) model based on 16 known binding sites of ZNF148 (Table 1)^4–6,8,42–50^ was applied on regions around known transcription starts (TS), following the approach described in Wasserman and Sandelin (2004). The PWM model slides across the region one nucleotide at a time, generating a score between 0 and 1 at each position for both strands. The best score (of the two strands) for each position was then averaged over a 200 bp window. A region was called as a potential binding site (PWM-peak positive) when the average score of the window exceeded 0.6.

### Meta-analysis of CRISPR and siRNA data

CRISPR-scores (Achilles_gene_effect), siRNA-scores (D2_combined_gene_dep_scores), and somatic mutation calls (CCLE_mutations) for human cancer cells (cancer cell line encyclopaedia) were downloaded from the Cancer Dependency Map portal at the Broad Institute (https://depmap.org/portal/download/).

### Drug response curves

15,000 cells were plated in 12-well plates (per well), incubated with cisplatin (Sigma), etoposide (Sigma), or TH588 (TH588 hydrochloride, Axon Medchem) for three days, and stained for 20 min with a fixing/staining solution containing 0.025% crystal violet (Sigma), 1% formaldehyde, and 1% methanol in PBS. After drying overnight, the crystal violet stained cells were solubilized in 10% acetic acid and the absorbance determined at 590 nm with a Spectramax II (Molecular Devices).

### Radiosensitivity

*Zfp148*^*gt/*+^ mice and wild-type controls were radiated using an RS-2000 X-ray irradiator (Rad Source Technologies) at the indicated doses. The thymus was collected after sacrifice on day 4 after radiation. Thymocytes were disseminated into single cell suspension and stained with CD45-BUV737 (clone 104, cat. no. 612778, BD Biosciences), CD4-BV785 (clone RM4-5, cat. no. 1102760, Sony Biotechnology), CD8-FITC (clone 53-6.7, cat. no. 1103525, Sony Biotechnology) and propidium iodide (cat. no. P3566, Life Technologies). Samples were run on a Fortessa X20 (BD Biosciences) and data was analyzed using FlowJo version 10.6.1.

### Statistical analyses

Values are mean ± SEM. Statistics were performed with Student’s *t*-test for comparisons between two groups; one-way ANOVA for multiple groups; Fisher exact test for contingency tables; two-sample Kolgomorov-Smirnov test for cumulative distribution function plots; Spearman correlation coefficient for ranked values; and Pearson correlation coefficient for continuous variables. Differences between groups were considered significant when *P* < 0.05.

## Supplementary information

Supplementary information 1.

Supplementary information 2.

Supplementary information 3.

Supplementary information 4.
