# Supplementary material for: Genomic profiling of the transcription factor Zfp148 and its impact on the p53 pathway
Source: Sci Rep. 2020 Aug 25;10:14156. doi: 10.1038/s41598-020-70824-2 (PMC7447789; doi:10.1038/s41598-020-70824-2)
Supplement: Supplementary file 1 — Supplementary information 1. [file 41598_2020_70824_MOESM1_ESM.pdf]

Supplementary Information

Genomic profiling of the transcription factor Zfp148 and its impact on the p53 pathway

Zhiyuan V. Zou[1], Nadia Gul[1,2,3], Markus Lindberg[4], Abdulmalik A. Bokhari[4], Ella M. Eklund[2,3], Viktor Garelick[1], Angana A.H. Patel[2,3], Jozefina J. Dzanan[2,3], Ben O. Titmus[2,3], Kristell Le Gal[2,3], Inger Johansson[1], Åsa Tivesten[1], Eva Forssell-Aronsson[x], Martin O. Bergö[5,6], Anna Staffas[4], Erik Larsson[4] ,Volkan I. Sayin[2,3*], and Per Lindahl[1,2*]

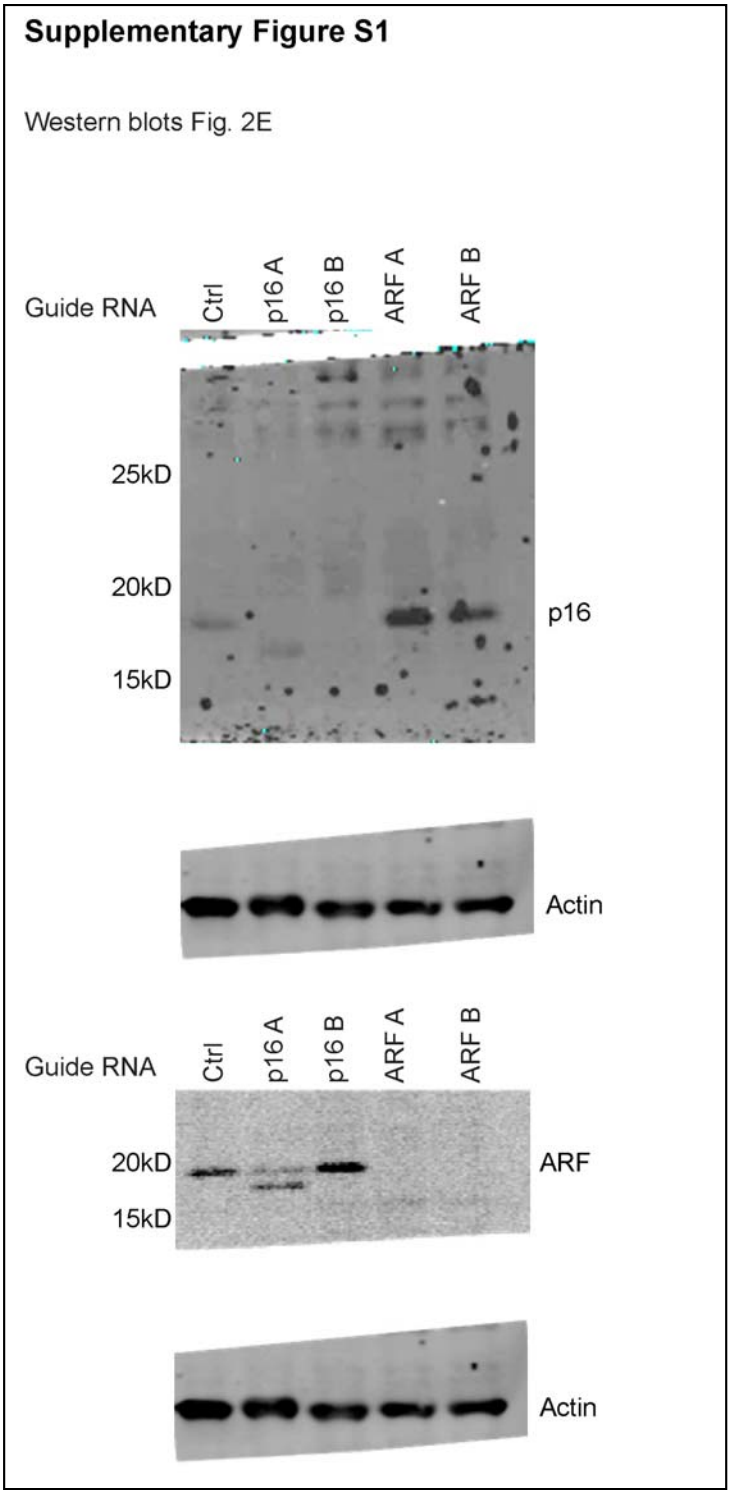

Supplementary Fig. S1. Uncropped western blot images in Fig. 2E. Western blots of protein extracts from *Zfp148*$^{gt/gt}$ MEFs infected with lentivirus expressing Cas9 and gRNA targeting p16, ARF, or negative control (Ctrl), with antibodies against p16 (top) or ARF (bottom). Actin was used as loading control.

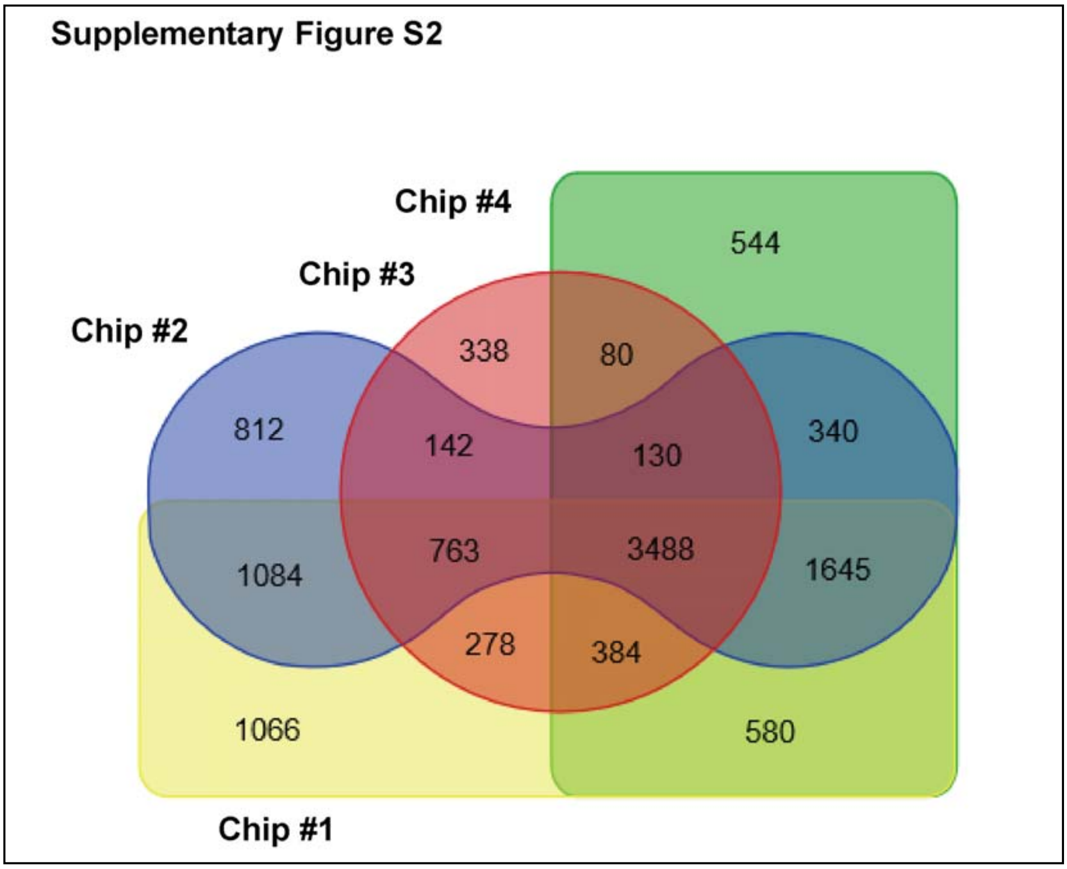

Supplementary Fig. S2. Venn diagram showing the overlap between individual ChIP-chip experiments.

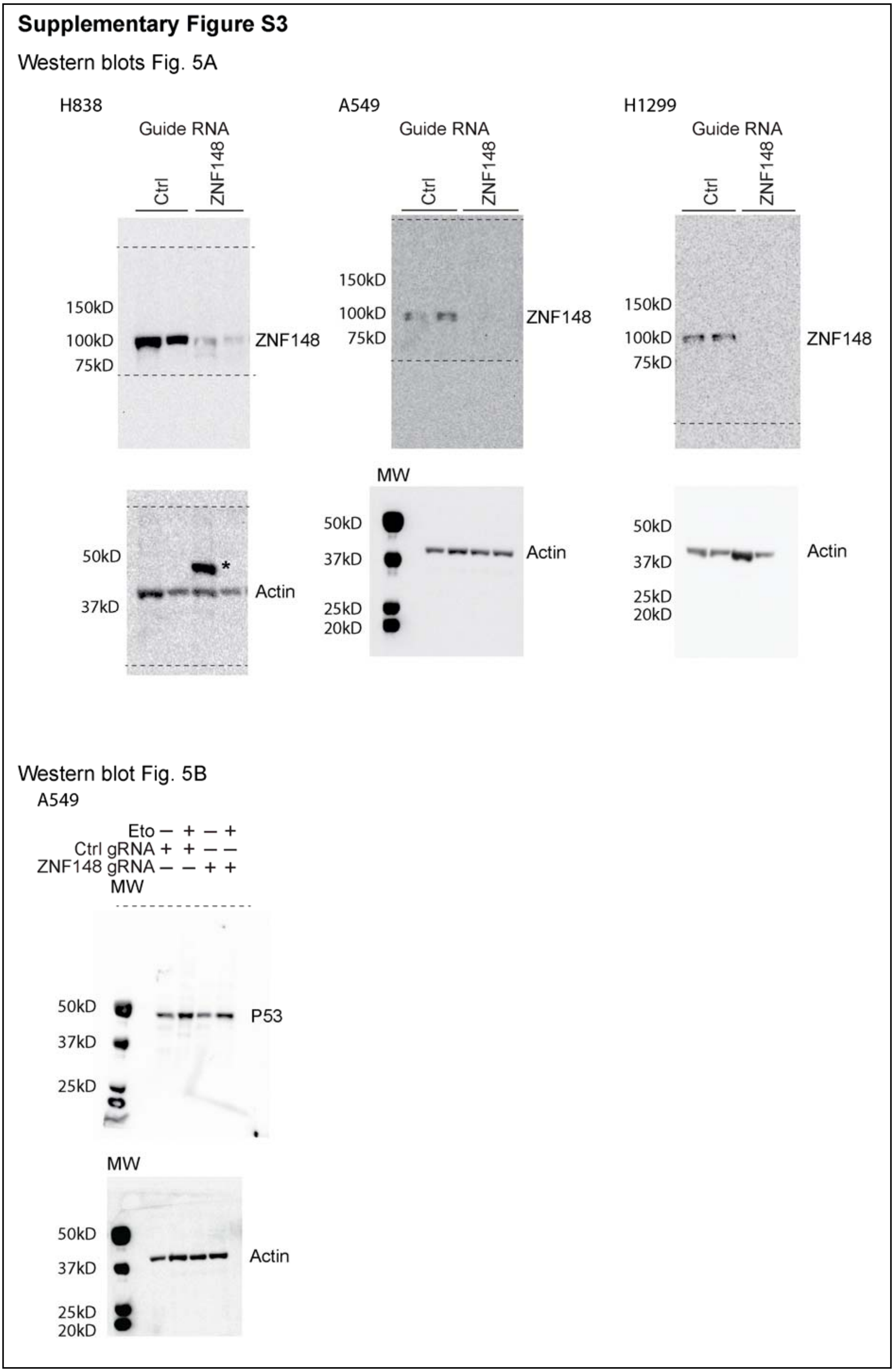

Supplementary Fig. S3. Uncropped western blot images in Fig. 5A and B. Western blots of

protein extracts from H838, A549, or H1299 cells infected with lentivirus expressing Cas9 and gRNA targeting *ZNF148* or negative control (Ctrl), with antibodies against ZNF148 (top) or p53 (bottom). Actin was used as loading control. *ectopic band; MW, molecular weight marker; Eto, etoposide. Dashed line indicates the border of the blot.
